# Stimulator of Interferon Genes (STING) Triggers Adipocyte Autophagy

**DOI:** 10.3390/cells12192345

**Published:** 2023-09-24

**Authors:** Kornél Z. Varga, Katalin Gyurina, Ádám Radványi, Tibor Pál, László Sasi-Szabó, Haidong Yu, Enikő Felszeghy, Tamás Szabó, Tamás Röszer

**Affiliations:** 1 Pediatric Obesity Research Division, Institute of Pediatrics, Faculty of Medicine, University of Debrecen, 4032 Debrecen, Hungary; 2 Institute of Neurobiology, Ulm University, 89081 Ulm, Germany

**Keywords:** adipocyte, inflammation, immunity, STING, interferons, mitochondria

## Abstract

Innate immune signaling in adipocytes affects systemic metabolism. Cytosolic nucleic acid sensing has been recently shown to stimulate thermogenic adipocyte differentiation and protect from obesity; however, DNA efflux from adipocyte mitochondria is a potential proinflammatory signal that causes adipose tissue dysfunction and insulin resistance. Cytosolic DNA activates the stimulator of interferon response genes (STING), a key signal transducer which triggers type I interferon (IFN-I) expression; hence, STING activation is expected to induce IFN-I response and adipocyte dysfunction. However, we show herein that mouse adipocytes had a diminished IFN-I response to STING stimulation by 2′3′-cyclic-GMP-AMP (cGAMP). We also show that cGAMP triggered autophagy in murine and human adipocytes. In turn, STING inhibition reduced autophagosome number, compromised the mitochondrial network and caused inflammation and fat accumulation in adipocytes. STING hence stimulates a process that removes damaged mitochondria, thereby protecting adipocytes from an excessive IFN-I response to mitochondrial DNA efflux. In summary, STING appears to limit inflammation in adipocytes by promoting mitophagy under non-obesogenic conditions.

## 1. Introduction

Obesity is associated with an inflammatory cytokine milieu in the adipose tissue that eventually abrogates insulin sensitivity and promotes beta cell death [1]. Obese adipocytes also produce large quantities of type I interferons, and a high-fat diet strongly induces the expression of a type I interferon receptor in the adipose tissue in mouse [2]. The excessive production of type I interferons is destructive for adipocytes and evokes cell death [3], and type I interferons may be responsible at least in part to the autoimmune component of obesity-associated metabolic diseases [4,5]. Interferons may damage the mitochondrial network and the capacity for fat oxidation and thermogenesis, thus triggering metabolic inflammation and insulin resistance [6,7,8]. 

However, constitutive interferon signaling is crucial for maintaining the expression of immune genes, and proinflammatory signaling and interferon-stimulated genes are necessary for physiological adipocyte development and functioning [2,9,10,11,12,13,14]. The overexpression of interferon beta (IFNβ), for instance, may protect adipocytes in obesity [13] and a lack of adipocyte interferon alpha and beta receptor subunit 1 (IFNAR1) worsens the metabolic effects of diet-induced obesity [2]. However, IFNAR1 deficiency improves glucose tolerance in diet-induced obesity [7]. In light of these findings, the balance of type I interferon synthesis appears to be a key checkpoint in adipocyte functioning and obesity development.

The stimulator of interferon response genes (STING) is a key signal transducer in the pathway, triggering type I interferon expression [15]. An activation of the STING pathway worsens obesity and abrogates the thermogenic program in adipocytes [16,17]. Cytosolic DNA sensors proteins activate STING in response to cytosolic DNA molecules, including DNA released from mitochondria [17,18]. In turn, the inhibition of DNA efflux from mitochondria into the adipocyte cytosol reduces obesity-associated inflammation and insulin resistance [17,19]. STING is hence considered as a proinflammatory trigger of adipose tissue dysfunction [16,17,20]. However, mice treated with the STING agonist 2′3′-cyclic-GMP-AMP (cGAMP) have improved metabolic performance [21], challenging the canonical view on STING function in obesity. 

These conflicting reports prompted us to explore further the role of STING activation in adipocytes. We found that STING stimulation triggered autophagy in adipocytes, a process that protects adipocytes from pro-inflammatory effects of cytosolic DNA.

## 2. Materials and Methods

### 2.1. Animals and Cells

We used 6-day-old (young) and 8-week-old (adult) male C57BL/6 mice (Charles River Laboratories, Wilmington, MA, USA), housed under SPF conditions. For a high-fat diet (HFD) feeding of adult mice, we used an HFD rodent from SSNIFF Spezialdiäten (Soest, Germany, E15725-347). Primary mouse preadipocytes from inguinal adipose tissue, epididymal adipose tissue and interscapular brown adipose tissue, hepatocytes and skeletal muscle cells were isolated by collagenase digestion and the separation of cell fractions, as described in [10,22,23]. To ensure the depletion of adipose tissue macrophages (ATMs) from the harvested preadipocytes, we used magnetic bead cell purification of the stromal cells with an antibody against the F4/80 antigen (Miltenyi Biotec, Bergisch Gladbach, Germany). Preadipocytes were maintained in high-glucose Dulbecco’s modified Eagle medium (DMEM) supplemented with L-glutamine and 20 μg/mL insulin (I9278, Merck, Rahway, NJ, USA). Adipocyte differentiation was stimulated by DMEM supplemented with 20 μg/mL insulin, 50 mM 3-isobutyl-1-methylxanthine, 1 mM dexamethasone and 1 mM rosiglitazone. Lipid content was labeled with Oil red O (BioGnost, Zagreb, Croatia). For histomorphometry of fat cells, we used Olympus CellSens Dimension image analysis software (Olympus, Tokyo, Japan).

### 2.2. Human Samples

Subcutaneous adipose tissue (groin region and abdominal fat depot) from human infants, adolescents and young adults were collected during elective surgery, as described in [10,24]. Patient body mass index (BMI) and BMI z-score were determined according to guidelines from the World Health Organization and have been validated by using population-specific BMI standard deviation scores (BMI-SDS), as described [24,25,26]. Human subcutaneous adipose tissue preadipocytes were harvested and cultured in vitro as described in [10,24]. For children included in the study, written informed consent was obtained from parents/guardians. 

### 2.3. Cell Treatment

STING was stimulated with 10 μg/mL cGAMP (InvivoGen, San Diego, CA, USA). Toll-like receptor 3 (TLR3) was stimulated with 10 ng/mL naked p(I:C) and Toll-like receptor 4 (TLR4) with 100 ng/mL LPS (Merck, Rahway, NJ, USA). As negative control, we used ssRNA (InvivoGen). STING was inhibited with the irreversible STING inhibitor H-151 (0.5 μM, InvivoGen) [27]. NFκB was inhibited with 5 μM BAY 11-7082 (Cayman Chemical Company, Ann Arbor, MI, USA). *Irf3* expression was suppressed via siRNA silencing, using oligos from Thermo Fisher (Waltham, MA, USA) (Silencer™ Pre-Designed siRNA, Cat. No.: AM16708, siRNA ID: 184585). Autophagy was stimulated with serum deprivation. Autophagy was inhibited with 100 μM chloroquine treatment, as described in [28].

### 2.4. Imaging of Mitochondria

For a fluorescent microscopy of mitochondrial content and morphology, preadipocytes were grown on optical transparent glass-bottom plates (Greiner Bio-One GmbH, Frickenhausen, Germany) or glass coverslips. Functional mitochondria were labeled with MitoTracker Red or its fixation-resistant substituent MitoBacon Orange (BioCat, Heidelberg, Germany). Mitochondria were also labeled with CellLight^TM^ Mitochondria-GFP BacMam 2.0 transfection system (LifeTechnologies Corporation, Eugene, OR, USA). Mitchondrial succinate dehydrogenase complex subunit A (SDH-A) and cytochrome c oxidase I (COX-I) level were measured with spectrophotometry (BioGnost enzyme cytochemistry kits) in cells cultured in 96-well plates, and SDH-A protein level was measured with ELISA (MyBioSource, Inc., Vancouver, BC, Canada). 

### 2.5. Autophagy Assays

Autophagosomes were labeled with Cell Meter^TM^ Autophagy Fluorescent Imaging Kit (AAT Bioquest, Sunnyvale, CA, USA) and photographed with an Olympus IX83 inverted fluorescent microscope. For a fluorimetric quantification of autophagy, we cultured cells in 96-well plates and stained them with a fluorescent Autophagy Assay Kit (MAK138-1KT, Merck), according to the manufacturer’s protocol. Autophagy-related gene product 5 (ATG5) and microtubule-associated protein 1A/1B-light chain 3 (LC3) were labeled with polylonal rabbit antibodies (Merck) in cells cultured on optical transparent glass-bottom plates (Greiner Bio-One GmbH, Frickenhausen, Germany), or quantified with an in-cell ELISA (BioCat, Heidelberg, Germany). AF488-conjugated secondary antibodies were used for visualization. Lysosomes were labeled with Lyso Brite Orange (Bertin Bioreagent, Montigny le Bretonneux, France) and Lyso View 405 (Biotium, Inc., Fremont, CA, USA) in cells cultured on optical transparent glass-bottom plates.

### 2.6. mRNA Analysis

Extraction of total RNA from adipose tissue or plasma was performed using TRIzol reagent (Merck Sigma Aldrich, St. Louis, MO, USA) as described in [29]. qPCR assays were carried out on a Quantabio platform (Beverly, MA, USA) and on an Analytik Jena platform, using the mean threshold cycle (CT) value for *Actinb*, *Gapdh* and *Ppia* (mouse) or *ACTINB* and *GAPDH* (human) as references. Primer sequences are shown in Table 1.

Protein–protein interactome maps were rendered with STRING [30]. Next-generation sequencing datasets are available in NIH GEO under accession number GSE154925, GSE185317, as described [24,29].

### 2.7. Histology, Immunofluorescence and Flow Cytometry

Tissues were fixed with 4% paraformaldehyde and embedded in paraffin. Sections were stained with hematoxylin and eosin (Carl Roth, Karlsruhe, Germany). STING and cGAS immunohistochemistry was performed on paraffin-embedded tissue sections, using a polyclonal antibody against mouse/human STING raised in rabbit (NBP2-24683, 1:250, Novus Biologicals, Denver, CO, USA or 13657, 1:250, Cell Signaling Technology, Danvers, MA, USA) or against mouse/human cGAS raised in rabbit (201708-T10, 1:125, Sino Biological, Eschborn, Germany). For fluorescent microscopy of STING and cGAS, murine or human preadipocytes were grown on optical transparent glass-bottom plates (Greiner Bio-One GmbH, Frickenhausen, Germany) or glass coverslips and labeled with the same antibodies used for immunohistochemistry, and then visualized with AF488-conjugated secondary antibody (Invitrogen, Carlsbad, CA, USA). Histology images were adjusted to equal white balance after acquisition. Flow cytometry analysis was used to detect STING^+^, cGAS^+^ macrophages and adipocytes, as described in [24]. Flow repository identifier of FACS data is FR-FCM-Z236. 

### 2.8. Western Blotting

Cells were lysed in ice-cold RIPA buffer supplemented with Pierce™ protease and phosphatase inhibitor mini tablets (Thermo Scientific). Protein concentration was measured using the Pierce™ Rapid Gold BCA Protein Assay Kit and 30–40 µg protein samples were run on 10% SDS gels for protein separation, followed by blotting the gels on 0.2 µm nitrocellulose blotting membrane (Amersham, Freiberg, Germany) at 300 mA for 1 h in a cold room. After blotting, membranes were blocked with 5% skimmed milk for 1 h. Antibody concentrations used were as follows: β-actin, 1:10,000 (NB600-532SS, Novus Biologicals); LC3, 0.2 µg/mL (L8918, Merck) [31]. 

## 3. Results

### 3.1. STING Is Constitutively Expressed in Adipocytes

**Figure 1:**
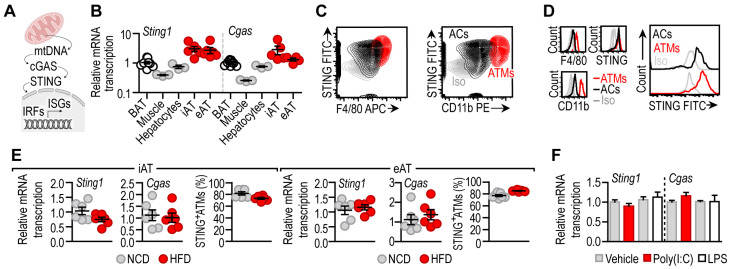
(**A**) Scheme summarizing the role of cGAS/STING in recognition of mitochondrial DNA (mtDNA) in the cytosol. (**B**) Transcript level of *Sting1* and *Cgas* in BAT, skeletal muscle, hepatocytes, iAT and eAT of male C57/BL6 mice at 8 weeks of age. Each data point represents one biological replicate. (**C**) FACS plot representing STING expression level of adipocytes (ACs) and adipose tissue macrophages (ATMs) of male C57/BL6 mice at 8 weeks of age. Extended analysis is presented in [24]. (**D**) Histograms comparing STING levels of ACs and ATMs. Iso: isotype control. Extended analysis in [24]. (**E**) Transcript levels of *Sting1* and *Cgas* in iAT and eAT of normal chow-diet (NCD)-fed or high-fat diet (HFD)-fed male C57/BL6 mice. Prevalence of STING^+^ ATMs in iAT and eAT, expressed as the percentage of the total ATM population. Extended analysis presented in [24]. Each data point represents one biological replicate. (**F**) Level of *Sting1* and *Cgas* mRNA in mouse adipocytes cultured in vitro and treated with 10 ng/mL poly(I:C) (a TLR3 ligand) or 100 ng/mL LPS (a TLR4 ligand) for 18 h.

The cyclic GMP-AMP synthase/stimulator of interferon genes (cGAS/STING) pathway is a relevant sensor of cytosolic DNA molecules and is an inducer of interferon responsive genes (ISGs) (Figure 1A). First, we measured *Sting1* (also known as *Tmem173*) mRNA—encoding STING protein—expression level in metabolically relevant tissues in mouse (Figure 1B, Supplemental Figure S1A). We found that *Sting1* was prominently expressed in inguinal and epididymal adipose tissue depots (iAT and eAT, respectively), while the interscapular brown adipose tissue depot (BAT) had a negligible basal expression of *Sting1* (Figure 1B). Skeletal muscle and hepatocytes had similarly low *Sting1* levels. 

We also measured the mRNA expression level of *Cgas* (also known as *Mb21d1*) encoding cGAS, the upstream activator of STING. Mirroring the expression pattern of *Sting1*, the expression of *Cgas* was prominent in iAT and eAT and was minimal in BAT, hepatocytes and skeletal muscle (Figure 1B). Adipocytes and adipose tissue macrophages (ATMs) equally expressed STING protein (Figure 1C,D) [24,29]. This finding reflected the single-cell sequencing data retrieved from the Tabula Muris Consortium database [32] and our previous findings [24]. These findings show that cGAS/STING signaling is ubiquitously expressed in white adipose tissue depots, unlike in the liver, where basal STING expression is confined to non-parenchymal cells [33]. 

Obesity is associated with changes in the cellular composition of the white adipose tissue and with a proinflammatory activation of both adipocytes and ATMs. Thus, we next asked whether diet-induced obesity affected *Sting1* and *Cgas* levels in the iAT and the eAT. Mice were fed with a high-fat diet (HFD) for 8 weeks, inducing adipose tissue inflammation [24], and the levels of *Sting1* and *Cgas* were measured in the iAT and the eAT. We found that HFD feeding did not alter *Sting1* and *Cgas* levels (Figure 1E). The amount of STING^+^ ATMs was also similar in lean mice and in obese mice fed with a HFD (Figure 1E). 

The stimulation of adipocytes with the TLR3 ligand polyinosine–polycytidylic acid (poly(I:C)) and the TLR4 ligand lipopolysaccharides (LPSs) triggers a pro-inflammatory gene expression (Supplemental Figure S1B), resembling the situation observed in the obese adipose tissue. However, treating adipocytes with poly(I:C) or LPS did not affect their *Sting1* and *Cgas* levels (Figure 1F). 

**Figure 2:**
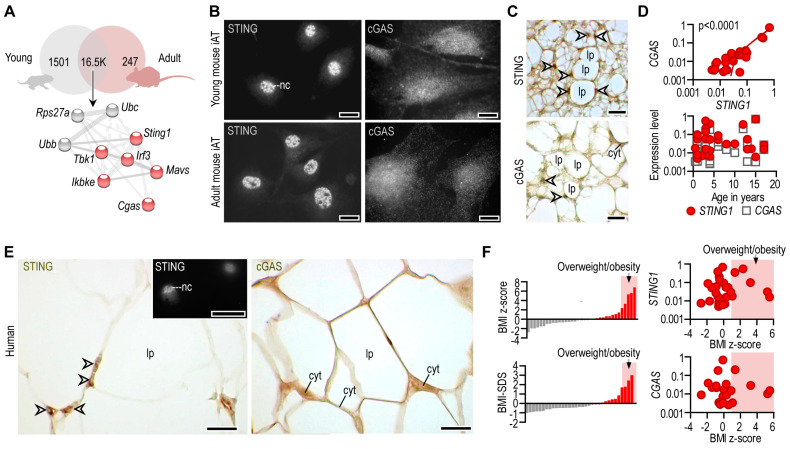
Expression of STING and cGAS mRNA and protein in adipocytes. (**A**) Venn diagram summarizing the number of equally and differently expressed mRNA transcripts of young and adult mouse iAT. A gene network associated with *Sting1* was equally expressed by young and adult iAT. A protein–protein interaction map, generated by STRING [30] is shown below the Venn diagram. Extended analysis presented in [24]. (**B**) Immunofluorescence of in vitro cultured adipocytes from young and adult mouse iAT; nc: nucleus, scale bar 20 μm. (**C**) Immunostaining of STING and cGAS proteins in the iAT of young mice, showing a region containing both multilocular and unilocular adipocytes. Arrowheads label nuclei; lp: lipid droplet; cyt: cytoplasm; scale bar: 50 μm. (**D**) *Top:* Expression of *STING1* and *CGAS* mRNA in human inguinal and abdominal adipose tissue specimens. Linear regression analysis indicates a significant positive correlation between *STING1* and *CGAS* mRNA levels. Each data point represents one tissue donor patient. *Bottom:* Correlation of donor age and the adipose tissue expression levels of *STING1* and *CGAS*. (**E**) Immunohistochemistry of STING and cGAS proteins in human adipose tissue, collected from the inguinal-low abdominal region. Nineteen-month-old male infant; arrowheads label nuclei; lp: lipid droplet; cyt: cytoplasm; scale bar: 25 μm. Inlet shows nuclear STING labeling of an in vitro cultured human adipocyte. Scale bar: 20 μm. (**F**) Body mass index z-score (BMI z-score) and BMI standard deviation score (BMI-SDS) of adipose tissue donors involved in this study. Correlation of BMI z-score with adipose tissue *STING1* and *CGAS* mRNA levels.

Next, we asked whether the expression of the STING/cGAS signaling was sensitive to the developmental stage of the white AT. We measured the level of *Sting1* and *Cgas* in the iAT of young (postnatal day 6) and adult (8 weeks of age) mice (Figure 2A). Since eAT is not relevant in young mice, we could not assess this depot at postnatal day 6. The level of *Sting1* and *Cgas* was similar in young and adult iAT; moreover, the gene network associated with *Sting1* was equally expressed by young and adult iAT (Figure 2A). 

Coherently, STING and cGAS proteins were expressed at similar levels in adipocytes of young and adult mice (Figure 2B). STING was closely associated with the cell nuclei, while cGAS was distributed throughout the cytoplasm, with increased density in the perinuclear compartment (Figure 2B). A nuclear and perinuclear localization of STING and cGAS was seen in tissue sections of mouse iAT as well (Figure 2C). STING and cGAS were present in both multilocular and unilocular adipocytes (Figure 2C). 

We next analyzed human subcutaneous adipose tissue specimens that were removed from the abdominal and groin regions during elective surgery, as described in [24]. There was a strong positive correlation between *STING1* and *CGAS* mRNA levels in human subcutaneous adipose tissue, and *STING1* levels mirrored *CGAS* levels during postnatal fat development (Figure 2D). Such as in mice, we found a perinuclear and cytosolic distribution pattern of STING and cGAS proteins in human subcutaneous adipose tissue (Figure 2E).

Adipocytes—similarly to almost all somatic cells—shed extracellular vesicles into the bloodstream, and these vesicles contain RNA cargo. We found that human plasma contained *STING1* mRNA (Supplemental Figure S2A). Adipose tissue levels of *STING1* and *CGAS* were unaffected by obesity status, albeit plasma *STING1* level was moderately increased with increasing BMI z-score (Figure 2F, Supplemental Figure S2B). Plausibly, this was due to the increased fat mass, leading to an increased level of adipocyte-derived mRNA cargo in the blood plasma.

Altogether, STING1 and CGAS were constitutively expressed in the adipose tissue of mice and humans. The developmental stage of adipocytes, obesity status and inflammatory signals did not correlate with the expression of the cGAS/STING pathway in the adipose tissue. 

### 3.2. IFN-I Response following STING Activation in Adipocytes

**Figure 3:**
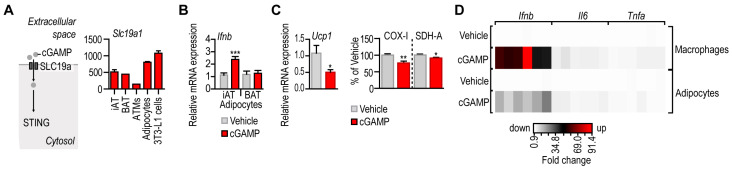
Response of mouse adipocytes and macrophages to a STING ligand. (**A**) *Left:* Uptake route of the STING ligand 2′3′-cyclic-AMP-GMP (cGAMP). Membrane transport of cGAMP is facilitated by the solute carrier protein SLC19a. *Right:* Expression of *Slc19a1* mRNA in mouse iAT, BAT, ATMs, primary adipocytes and in mouse 3T3-L1 cells. Secondary NGS analysis from [29]. (**B**) *Ifnb* expression level in iAT- and BAT-derived adipocytes, treated in vitro with 10 µg/mL cGAMP for 18 h. (**C**) Expression of *Ucp1* mRNA and the activity of mitochondrial enzymes COX-I and SDH-A in mouse iAT-derived adipocytes treated with cGAMP for 18 h. (**D**) Heat map summarizing the transcriptional changes of *Ifnb*, *Il6* and *Tnfa* in mouse macrophages and adipocytes, following treatment with 10 µg/mL cGAMP for 18 h. * *p* < 0.05, ** *p* < 0.01, *** *p* < 0.001, Student’s unpaired 2-tailed *t*-test.

STING signaling is activated by a synthetic ligand, so-called 2′3′-cyclic-AMP-GMP (cGMP). Cellular uptake of cGAMP is facilitated by the solute carrier SLC19A1 (Figure 3A) [34]. To assess whether adipocytes and ATMs were capable of cGAMP uptake, we measured the transcript level of *Slc19a1* in iAT, BAT, adipocytes and ATMs isolated from iAT, and in the adipogenic mouse 3T3-L1 cell line. *Slc19a1* was expressed by all the tested tissues and cells, having the highest *Slc19a1* mRNA level in primary adipocytes and 3T3-L1 cells (Figure 3A). In BAT, however, the expression of *Sting1* and *Cgas* was much lower than in other fat depots (Figure 1B). Accordingly, cGAMP treatment failed to trigger *Ifnb* expression in BAT-derived adipocytes (Figure 3B), suggesting a lack of functional STING signaling in brown adipocytes. It has been shown that STING activation inhibits thermogenic adipocyte development [19]. Adipocytes of iAT in young mice are thermogenic [10,24] and express both *Sting1* and *Cgas* mRNA [29]. We next treated the adipocytes of young mice with cGAMP. As expected, this treatment diminished the expression of *Ucp1*, encoding uncoupling protein 1, a major thermogenic protein (Figure 3C). Moreover, cGAMP also diminished mitochondrial enzyme activities in adipocytes (Figure 3C).

Altogether, adipocytes with a concomitant expression of *Sting1*, *Cgas* and *Slc19a1* responded to cGAMP treatment with *Ifnb* expression, diminished *Ucp1* level and reduced mitochondrial enzyme activities. This accords to previous observations on the role of STING in white adipose tissue [16,17,20]. However, cGAMP triggered a less robust interferon response in white adipocytes than in macrophages (Figure 3D), suggesting that white adipocytes had a mechanism that mitigated a STING-induced interferon response.

### 3.3. STING Activation Triggers Mitophagy in Adipocytes

Mitochondria are the main sources of cytosolic DNA [35], and mitochondrial DNA is a potent trigger of STING-mediated interferon response [36]. The removal of aged or damaged mitochondria via mitophagy protects the cytosol from an efflux of mitochondrial DNA into the cytosol [36,37,38]. Mitophagy cooperates with mitochondrial fusion and fission to ensure the quality control of mitochondria [37], and mitochondrial fusion activates STING [39]. In turn, in some cells, STING activation increases autophagic flux through its interaction with microtubule-associated protein 1A/1B-light chain 3 (LC3) [40], autophagy-related gene product 5 (ATG5) and through the phosphorylation of mitophagy adaptors [41]. Since mitophagy is a potential mechanism that mitigates interferon response, we turned our attention to a possible autophagy-inducing effect of STING.

**Figure 4:**
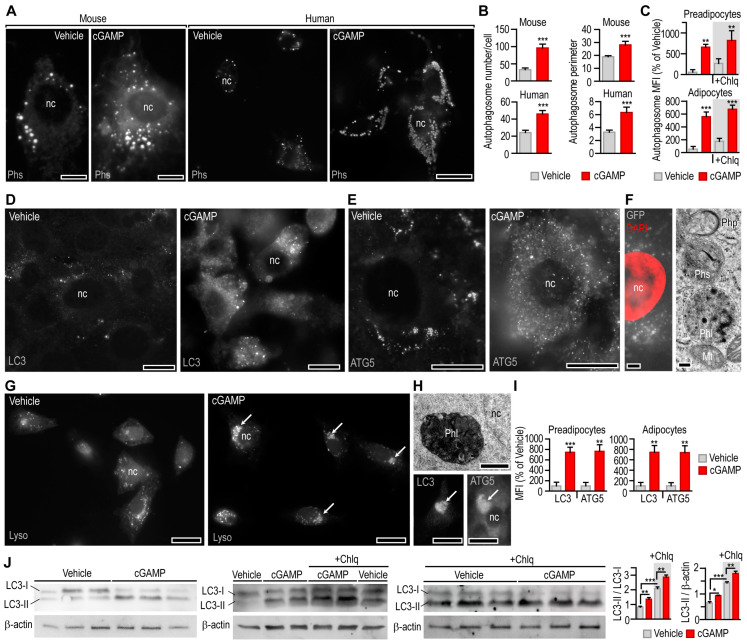
Effect of cGAMP on autophagy in adipocytes. (**A**) Autophagosomes (Phs) were labeled with a CellMeter^TM^ autophagy fluorescent imaging probe in primary mouse and human adipocytes. Adipocytes were treated with vehicle or 10 µg/mL cGAMP for 6 h. (**B**) Phagosome number and perimeter in vehicle-, or cGAMP-treated mouse and human adipocytes. (**C**) A fluorescent autophagy assay was used to estimate autophagosome number in mouse 3T3-L1 preadipocytes and adipocytes following treatment with vehicle or cGAMP. +Chlq: cells were also treated with 100 μM chloroquine for 4 h. (**D**,**E**) LC3^+^ and ATG5^+^ puncta in mouse adipocytes treated with vehicle or cGAMP. nc: nucleus, scale bar: 20 μm. Corresponding staining of preadipocytes is shown in Supplementary Figure S3C. (**F**) Mitochondria were labeled with BacMam 0.2 transfection system. GFP-labeled mitochondrial remnants were accumulated in autophagosomes of cGAMP-treated adipocytes. nc: nucleus, scale bar: 10 μm. Adipocytes were treated with 10 µg/mL cGAMP for 6 h. Transmission electron microscopy of phagophore (Php), phagosome (Phs), phagolysosome (Phl) and mitochondria (Mt). Scale bar: 0.1 μm. (**G**) Lysosomes (Lyso) were labeled with Lyso Brite Orange in human adipocytes and treated with vehicle or 10 µg/mL cGAMP for 2 h. Arrows indicate clustering of lysosomes. Scale bar: 20 μm. (**H**) Transmission electron microscopy of phagolysosome (Phl) and fluorescent microscopy of LC3^+^ and ATG5^+^ structures in mouse adipocytes. Adipocytes were treated with vehicle or 10 µg/mL cGAMP for 6 h. nc: nucleus. Scale bar: 0.5 μm (electron microscopy) and 10 μm (fluorescent microscopy). (**I**) Mean fluorescence intensity (MFI) of LC3 and ATG5 immunostaining in mouse preadipocytes and adipocytes treated with vehicle or 10 µg/mL cGAMP for 6 h. (**J**) Western blotting of LC3 in mouse adipocytes. Cells were treated with vehicle or cGAMP for 6 h. +Chlq: cells were also treated with 100 μM chloroquine for 4 h. * *p* < 0.05, ** *p* < 0.005, *** *p* < 0.001, two-tailed unpaired Student *t*-test (**B**,**I**) or one-way ANOVA with Dunnett’s post hoc test (**C**,**J**).

We found that cGAMP increased the number of autophagosomes in mouse and human primary adipocytes (Figure 4A,B). Autophagosome size increases during macroautophagy that is associated with the engulfment of damaged cell organelles, such as mitochondria [38]. The analysis of phagosome size indicated a cGAMP-induced increase in the phagosome perimeter (Figure 4B). The effect of cGAMP on phagosome number was apparent in both preadipocytes and in vitro differentiated adipocytes and was not sensitive to chloroquine treatment (Figure 4C). BAT was lacking *Sting1* expression, and cGAMP did not induce autophagy in BAT-derived adipocytes (Supplemental Figure S3A).

Chloroquine—similarly to genetic deficiencies in autophagosome function—blocks autophagosome–lysosome fusion, hence leading to the accumulation of autophagosomes, without increasing autophagic flux [42,43]. Indeed, chloroquine treatment increased autophagosome number (Figure 4C) and LC3 level in mouse adipocytes (Supplemental Figure S3B). Our finding on a chloroquine-resistant effect of cGAMP accords to previous findings, showing that STING increases autophagic flux and this effect is resistant to chloroquine [28]. Moreover, it has been shown that STING triggers autophagy by increasing the association of LC3 and ATG5 with autophagosomes [28,40]. Coherently, treatment with cGAMP increased the prevalence of LC3- and ATG5-positive puncta in preadipocytes and in adipocytes (Figure 4D,E, Supplemental Figure S3C). 

When mitochondria were labeled with green fluorescent protein (GFP), the GFP signal was enriched in phagosome-like puncta in response to cGAMP treatment (Figure 4F). Following cGAMP treatment, adipocyte lysosomes appeared in clusters, suggesting an increased rate of lysosome–autophagosome fusion (Figure 4G). Some of the LC3^+^ and ATG5^+^ structures resembled the morphology of autophagolysosomes (Figure 4H). Coherently, cGAMP increased LC3 and ATG5 levels in mouse preadipocytes and adipocytes (Figure 4I). Autophagosome formation is associated with a lipidation of LC3, and this LC3-phospholipid conjugate (LC3-II) is localized on autophagosomes [44,45]. Non-lipidated LC3 (LC3-I) and LC3-II have distinct molecular weights; hence, a Western blot detection of LC3-I and LC3-II is used to estimate autophagosome formation [44,45]. We found that cGAMP treatment increased LC3-II level compared to β-actin (Figure 4J). Moreover, the stimulation of LC3-I to LC3-II turnover by cGAMP was indicated by an increased ratio of LC3-II to LC3-I (Figure 4J). Chloroquine treatment similarly increased LC3-II level (Figure 4J). This is a known effect of chloroquine, and it appears due to the blocking of autophagosome–lysosome fusion and the eventually compromised lysosomal degradation of LC3-II. Treatment with cGAMP could overcome chloroquine effect and increase the ratio of LC3-II to LC3-I, and the ratio of LC3-II to β-actin (Figure 4J). 

These findings suggest that cGAMP increased autophagosome formation and allowed phagosome–lysosome fusion [42]. There are limitations of using LC3-II Western blotting to estimate autophagic flux [46], however, and changes in LC3-II level should be interpreted in context of other autophagy assays [47]. For instance, LC3-I is mostly cytosolic [48], while LC3-II is associated with autophagosomes [49]; thus, detecting subcellular LC3 distribution is necessary to complement Western blot findings. Our Western blot findings reflect our microscopy observations with regard to increased autophagosome number and size, an increased number of LC3^+^ puncta, the clustering of lysosomes around autophagosomes, and the presence of autophagolysosomes in cGAMP-treated cells. Altogether, cGAMP appears to increase autophagic flux. 

**Figure 5:**
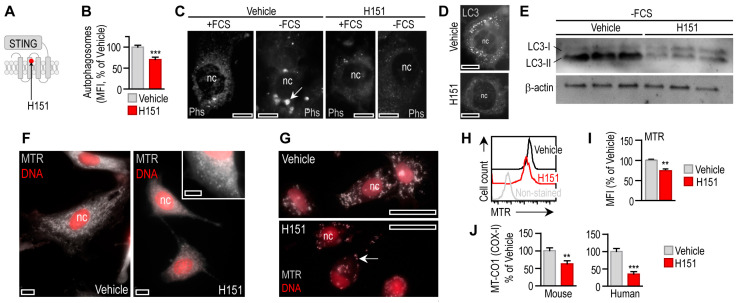
Effect of STING blockage on adipocyte autophagy. (**A**) H151 covalently binds to STING [27]. (**B**) Autophagy intensity in mouse adipocytes treated with vehicle or 0.5 μM H151 for 18 h. (**C**) Fluorescently labeled autophagosomes (Phs) in mouse adipocytes cultured in the presence of fetal calf serum (+FCS) or serum-deprived (−FCS) for 6 h. Cells were treated with vehicle or H151 for 6 h. (**D**) LC3 immunostaining of mouse adipocytes treated with vehicle of H151 during 6 h serum deprivation. Scale bar: 10 μm. (**E**) LC3 Western blot of mouse adipocytes following 6 h serum deprivation. Cells were treated with vehicle or H151 during serum deprivation. (**F**) Mitochondrial network of mouse adipocytes was labeled with MitoTracker Red (MTR) and treated with vehicle or H151 for 18 h. Scale bar: 5 μm. (**G**) Mitochondrial network of human adipocytes was labeled with MTR and treated with vehicle or H151 for 18 h. Arrow labels mitochondria. Scale bar: 20 μm. (**H**,**I**) FACS analysis and MFI of MTR labeling of mouse adipocyte mitochondria after 18 h H151 treatment. (**J**) Activity of mitochondrial COX-I in mouse and human adipocytes, following treatment with vehicle or H151 for 18 h. ** *p* < 0.01, *** *p* < 0.001. Student’s unpaired 2-tailed *t*-test.

Our observations indicate that STING activation triggered autophagy in adipocytes. In turn, when we blocked STING signaling with a synthetic covalent inhibitor, so-called H151 [27] (Figure 5A), the autophagy activity of adipocytes was reduced (Figure 5B). We triggered autophagy with serum deprivation, leading to the increase in autophagosome number (Figure 5C). However, H151 treatment abrogated autophagy in serum-deprived cells (Figure 5C). Coherently, H151 also inhibited the formation of LC3^+^ puncta (Figure 5D). Unlike genetic mutations leading to the accumulation of non-functional autophagosomes [43], H151 blocked autophagosome biogenesis. H151 also blocked serum deprivation-induced LC3-I shift to LC3-II, indicating a blockage in autophagic flux (Figure 5E). 

Impaired autophagy impairs mitochondrial quality [36,50]. Coherently, the number of functional mitochondria was decreased by H151 (Figure 5F–I), along with compromised mitochondrial enzyme activities (Figure 5J). Similar to mitophagy, mitochondrial fusion and fission are necessary processes for mitochondrial quality control, and have their specific effects on the interferon response [51]. For instance, deficiency in mitofusin 1—an effector in mitochondrial fusion—inhibits STING signaling and interferon response [39]. In turn, STING induces mitochondrial fusion in a pancreatic cancer cell line [52]. H151 treatment increased the accumulation of the mitochondrial protein SDH-A and the mitochondrially encoded transfer RNA *TrnQ* (Supplemental Figure S3D), despite the decrease in the number of functional mitochondria and suppressed mitochondrial respiration (Figure 5F–J). These findings suggest that H151 decreased the autophagic removal of mitochondria. 

**Figure 6:**
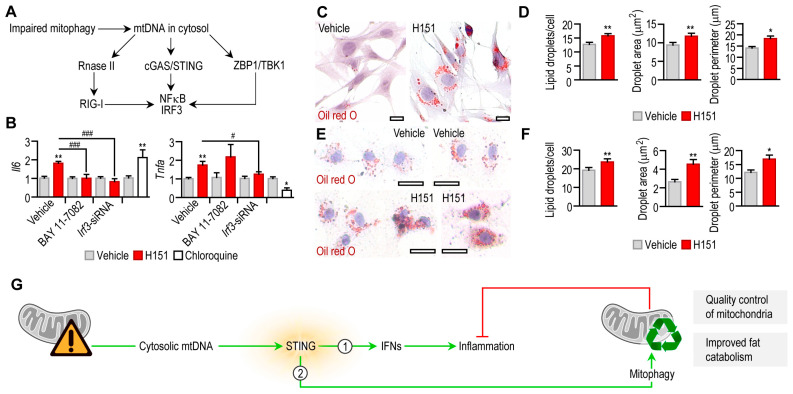
Effect of STING inhibition on the inflammatory state and lipid accumulation of adipocytes. (**A**) Scheme of cytosolic DNA sensor pathways that respond to mitochondrial DNA (mtDNA). (**B**) Expression levels of *Tnfa* and *Il6* mRNA in mouse adipocytes treated with vehicle or 0.5 μM H151 for 18 h. Cytosolic DNA sensors pathways were blocked by the NFκB inhibitor BAY 11-70082, or by transfecting cells with an *Irf3*-siRNA. As a comparison, adipocytes were treated with vehicle or 100 μM chloroquine for 18 h. (**C**) Oil red O labeling of lipid droplets in mouse preadipocytes, treated with vehicle or H151 for 18 h. Scale bar: 20 μm. (**D**) Number, area and perimeter of lipid droplets following treatment with vehicle or H151 for 6 h. (**E**) Oil red O labeling of lipid droplets in human preadipocytes, treated with vehicle or H151 for 18 h. Scale bar: 20 μm. (**F**) Number, area and perimeter of lipid droplets in human preadipocytes following treatment with vehicle or H151 for 6 h. * *p* < 0.05, ** *p* < 0.01, Student’s unpaired 2-tailed *t*-test; # *p* < 0.05, ### *p* < 0.001, one-way ANOVA with Dunnett’s post hoc test. (**G**) Working model summarizing the dual roles of STING in adipocytes. STING activation promotes expression of interferons (IFNs) and causes inflammation. In turn, an anti-inflammatory effect of STING exists in adipocytes by increasing autophagic removal of inflammation-provoking mitochondrial contents. Also, STING appears to control lipid content in adipocytes.

Impaired mitophagy leads to the accumulation of mitochondrial nucleic acids in the cytosol, which triggers inflammation and damages mitochondria secondarily [36,50]. We next tested whether H151 caused inflammation. Cytosolic nucleic acid sensor proteins, such as retinoic acid-inducible gene I (RIG-I), Z-DNA binding protein (ZBP1) and cGAS/STING may be stimulated by mitochondrial nucleic acids (Figure 6A) [16]. 

Blocking STING with 0.5 μM H151 induced *Tnfa* and *Il6* transcription in mouse adipocytes (Figure 6B). This effect was diminished or lacking when nuclear factor kappa B (NFκB) or interferon regulatory factor 3 (IRF3)—both pro-inflammatory effectors in cytosolic nucleic acid sensing pathways—was inhibited, respectively (Figure 6B). Chloroquine mirrored the effect of H151 by increasing *Il6* transcription (Figure 6B). Increased IL-6 secretion is known to further inhibit starvation-induced autophagy [53]. However, chloroquine blocks proinflammatory cytokine secretion [54], and accordingly, it abrogated *Tnfa* transcription (Figure 6B). These findings confirm that impaired mitophagy by the blockage of STING provoked inflammation in adipocytes, plausibly due a diminished mitophagy. 

Autophagy is key for adipocyte development and the regulation of lipid droplet volume in adipocytes [10,55,56]. The blockage of STING not only impaired the mitochondrial quality, but also caused lipid accumulation in both human and mouse adipocytes (Figure 6C–F). 

## 4. Discussion

Cytosolic nucleic acid sensing is a key mechanism that maintains cell integrity by triggering innate immune response toward foreign DNA or RNA of pathogens and nucleic acids released by damaged mitochondria or nucleus [57]. However, the resulting immune response may cause pyroptotic cell death [58], block autophagic removal of damaged mitochondria [59], limit thermogenic potential of the fat cells [19] and lead to a fulminant interferon response [16,57]. This causes inflammation and tissue damage and may also lead to autoimmunity [16,57,60].

In the obese adipose tissue, for instance, metabolic inflammation may be initiated through the stimulation of the cGAS/STING signaling by mitochondrial DNA of adipocytes [16,17,19]. Nucleic acid immunity hence may be detrimental for adipose tissue metabolism and evoke chronic inflammation, a key mechanism leading to obesity-associated diseases [16,57,60]. 

A relevant paradigm in obesity management is to reduce metabolic inflammation, and inhibiting nucleic acid immune signaling is thought to serve this need. However, recently, we have shown that mitochondrial RNA molecules serve as intracellular signal molecules in the developing adipocytes [24]. In brief, mitochondrial RNAs stimulate the RIG-I/MDA5 cytosolic nucleic acid sensor pathway and promote the expression of nuclear-encoded genes of mitobiogenesis and thermogenesis through an autocrine IL-6 loop [24]. IL-6 is known to be produced by preadipocytes and to stimulate the thermogenic fat differentiation [10,61]. Mitochondrial RNA sensing hence stimulates fat catabolism [24]. However, an overstimulation of this signal mechanism may lead to a detrimental loss of fat reserves [62]. Albeit interferons and IL-6 are necessary for early adipocyte development, the excessive production of interferons and IL-6 may either cause a cachectic loss of fat mass—lipodystrophy—or aggravate metabolic inflammation [63]. It is still to be explored how interferon and IL-6 production are controlled in the developing adipose tissue.

Here, we show that the STING pathway, a major stimulator of interferon response toward cytosolic DNA molecules, plays a dual role in adipocytes. It stimulates interferon and IL-6 synthesis; however, it also initiates autophagy (Figure 6G). Autophagy may antagonize cell death and may also help to clear the cytosol from damaged, potentially apoptosis-inducing, and pro-inflammatory mitochondria [64]. Mitochondrial contents, including RNA and DNA molecules, are inflammation provoking, damage-associated molecules and may exacerbate inflammation in the adipose tissue through various signal mechanisms, including the STING pathway [65]. 

Mitochondrial DNA is recognized by various cytosolic DNA sensors, such as the Z-DNA binding protein ZBP1 (also known as DAI) [66], interferon inducible protein 204 (IFI204), ATP-dependent RNA helicase DDX41 and AIM2 [24]. The most relevant DNA recognition system is the cGAS/STING pathway. We have shown previously that mitochondria-rich thermogenic adipocytes express less ZBP1, IFI204, DDX41 and AIM2 than their fat-storing counterparts [24]. A lack of these DNA sensors is protective from IFN-I response [67]. This suggests that the abundance of mitochondria is associated with a reduced expression of cytosolic DNA sensors. It may protect cells from an excessive interferon response to leaked mitochondrial DNA [24]. 

The expression levels of cGAS and STING, however, were constitutive during postnatal fat development. We found here that neither obesity nor inflammation affected cGAS and STING expression levels in adipocytes. This makes it plausible that an increase in mitochondrial mass—for instance, during adipocyte “browning”, i.e., the acquisition of thermogenic potential—may increase the probability of cGAS/STING activation through mitochondrial DNA. In obesity, mitochondrial damage and the leak of mitochondrial DNA into the cytosol are more prevalent than in the lean state. This condition also increases the probability of activating cGAS/STING signaling, causing inflammation. Contrary to our expectation, however, when STING was stimulated, autophagosome number and LC3-II level were increased in adipocytes, showing an increased autophagy. In turn, STING blockage reduced autophagosome number and LC3 level in adipocytes. 

It is known that STING is an autophagy-promoting molecule that increases autophagic flux [68], and mitophagy inhibition and impaired mitochondrial dynamics trigger STING activation and inflammation [69]. Indeed, the evolutionarily conserved role of STING is to initiate the formation of autophagosomes [28,40]. It is known that STING physically interacts with proteins necessary for autophagosome biogenesis and autophagosome–lysosome fusion, such as syntaxin-17 protein [68], WIPI2 (a WD-repeat PtdIns(3)P effector protein) [70] and LC3 [40]. Autophagy in adipocytes may limit the size of lipid droplets—in the process of lipophagy [56]—and remove damaged and inflammation-provoking mitochondria in the process of mitophagy [37]. Accordingly, limited autophagic competence makes mice vulnerable to diet-induced obesity and diabetes [71]. In turn, the stimulation of autophagy protects mice from these metabolic alterations [71]. Altogether, STING activation appears to protect from fat accumulation and support mitochondrial functioning in adipocytes.

STING is an important nucleic acid sensor that stimulates innate immune response to pathogens. Infectious diseases, host–pathogen, host–parasite and host–symbiont interactions have shaped the human metabolism over the course of evolution [72]. DNA viruses directly activate cGAS/STING signaling, and some RNA viruses induce mitochondrial DNA leakage into the cytosol and trigger STING signaling secondarily (reviewed by [67]). Viral infections deplete fat reserves stored in adipocytes and may even cause energy deficit by excessive thermogenesis from stored fat [62,73]. However, certain pathogens block STING activity [67] and some viruses increase mitophagy as an immune evasion mechanism to inhibit STING activation by mitochondrial nucleic acids [51]. Our findings suggest that STING blockage damages adipocyte energy production and may lead to excessive fat storage. Infections may lead to metabolic dysfunction and can have a lasting impact on endocrinology and metabolism. For instance, antiviral innate immune signaling can deteriorate insulin secretion and insulin signaling and can cause childhood obesity and diabetes [74,75,76,77,78] through yet largely unexplored mechanisms. This so-called infectobesity theory explains obesity as a metabolic response to early life infections. Albeit not explored here, our findings suggest a possible obesity-inducing effect of STING blockage by pathogens. 

## 5. Conclusions

We found that the inhibition of STING led to the blockage of autophagy, compromised the mitochondrial network and increased inflammatory gene expression and lipid droplet volume. These effects coherently show that STING was necessary for autophagy in adipocytes, mirroring STING functions known in other cell types. STING also affects mitochondrial fusion in some cell types, and further studies may address whether an equivalent role for STING exists in adipocytes. An additional effect on mitochondrial fusion thus may not be ruled out; nevertheless, STING inhibition led to the accumulation of non-functional mitochondria and mitochondrial contents.

Autophagy-promoting STING signaling appears to be part of a complex negative feedback mechanism that controls interferon-stimulated gene expression. For instance, STING stimulates the expression of IL-6 and RIG-I [79], and in turn, both IL-6 and RIG-I activation promotes STING degradation [79]. Downstream to STING, TANK binding kinase 1 (TBK1) phosphorylates NFκB and IRF3 and stimulates gene expression. TANK, however, inhibits the cGAS-dependent recognition of cytosolic DNA [80]. When autophagy is deficient, damaged mitochondria accumulate in the cells, causing inflammation through STING-independent DNA-sensing pathways [81]. Autophagy is hence necessary to limit STING-induced inflammation. 

In summary, our findings show that STING is important for DNA-induced non-canonical autophagy in adipocytes, and it limits fat accumulation and supports the turnover of the mitochondrial network.

## Figures and Tables

**Table 1:** Mouse and human qPCR primer sequences used in the study.

*Actinb*	fw	GCACCAGGGTGTGATGGTG
	rev	CCAGATCTTCTCCATGTCGTCC
*Ppia*	fw	ATTTCTTTTGACTTGCGGGC
	rev	AGACTTGAAGGGGAATG
*Gapdh*	fw	TGACGTGCCGCCTGGAGAAA
	rev	AGTGTAGCCCAAGATGCCCTTCAG
*Cgas/Mb21d1*	fw	AGGAAGCCCTGCTGTAACACTTCT
	rev	AGCCAGCCTTGAATAGGTAGGTAGTCCT
*Sting1/Tmem173*	fw	GGGCCCTGTCACTTTTGGTC
	rev	GAGTATGGCATCAGCAGCCAC
*Il6*	fw	GCTACCAAACTGGATATAATCAGGA
	rev	CCAGGTAGCTATGGTACTCCAGAA
*Tnfa*	fw	TGCCTATGTCTCAGCCTCTTC
	rev	GAGGCCATTTGGGAACTTCT
*Ifnb*	fw	CCAGCTCCAAGAAAGGACGA
	rev	CGCCCTGTAGGTGAGGTTGAT
*CGAS*	fw	CATGGCGGCTATCCTTCTCT
	rev	AAAGCAGAGGCCCAGGTCTT
*STING1*	fw	ATATCTGCGGCTGATCCTGC
	rev	GGTCTGCTGGGGCAGTTTAT
*GAPDH*	fw	GTCTCCTCTGACTTCAACAGCG
	rev	ACCACCCTGTTGCTGTAGCCAA
*ACTINB*	fw	CACCATTGGCAATGAGCGGTTC
	rev	AGGTCTTTGCGGATGTCCACGT
*TrnQ*	fw	GATGTCAGAGGGGTGCCTTG
	rev	AACCCTCGTTCCACAGAAGC

## Data Availability

NGS data, digital images and FACS data presented in this study are openly available in GEO (under accession numbers GSE154925, and GSE185317), Figshare (DOI: 10.6084/m9.figshare.24186915) and Flow Repository (under project number FR-FCM-Z236).

## Supplementary Materials

The following supporting information can be downloaded at: https://www.mdpi.com/article/10.3390/cells12192345/s1, Figure S1: (A) Scheme summarizing the anatomical sites of adipose tissues used in this study; (B) Expression of proinflammatory genes in mouse adipocytes treated with poly(I:C) or LPS for 18 h; Figure S2: (A) Correlation of BMI z-score and plasma level of STING1 mRNA in human subjects; (B) Correlation of BMI standard deviation score (BMI-SDS) with adipose tissue expression level of *STING1* and *CGAS* mRNA in human subjects; Figure S3: (A) Autophagosomes labeled with a fluorescent probe in BAT-derived mouse adipocytes treated with vehicle or 10 mg/ml cGAMP for 2 h; (B) Autophagosome mean fluorescence intensity in mouse preadipocytes treated with vehicle or 100 µM chloroquine for 4 h; (C) LC3 and ATG5 immunostaining of mouse primary preadipocytes treated with vehicle or cGAMP; (D) Level of mitochondrial succinate dehydrogenase A protein and mitochondrially encoded transfer RNA *TrnQ* in mouse preadipocytes treated with vehicle or H151 for 18 h.
