# Supplementary material for: Stimulator of Interferon Genes (STING) Triggers Adipocyte Autophagy
Source: Cells. 2023 Sep 24;12(19):2345. doi: 10.3390/cells12192345 (PMC10572001; doi:10.3390/cells12192345)
Supplement: Supplementary file 1 [file cells-12-02345-s001.zip › cells-2466461-supplementary.pdf]

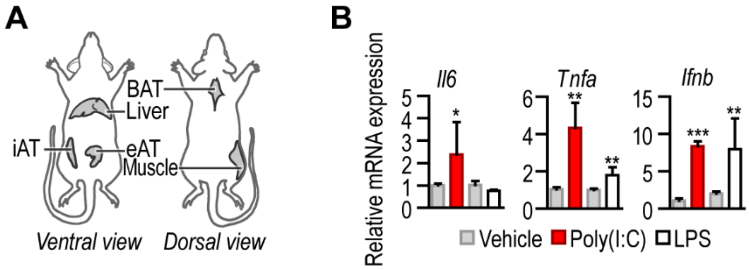

Supplemental Figure 1.
(A) Scheme summarizing the anatomical sites of adipose tissues used in this study. iAT: inguinal adipose tissue, eAT: epididymal adipose tissue, BAT: interscapular brown adipose tissue. (B) Expression of proinflammatory genes in mouse adipocytes treated with poly(I:C) or LPS for 18h. Corresponding experiment shown in Fig. 1F. *p<0.05, **p<0.005, ***p<0.001, Student's unpaired 2-tailed t-test.

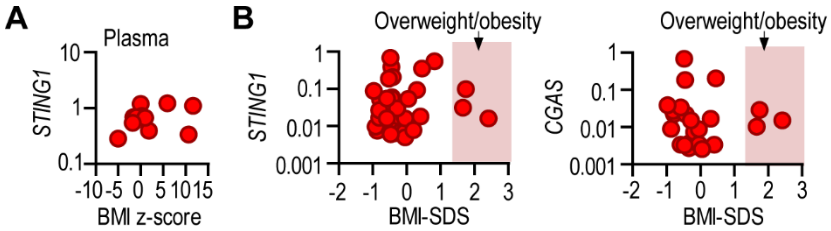

Supplemental Figure 2.
(A) Correlation of BMI z-score and plasma level of STING1 mRNA in human subjects. (B) Correlation of BMI standard deviation score (BMI-SDS) with adipose tissue expression level of *STING1* and *CGAS* mRNA in human subjects.

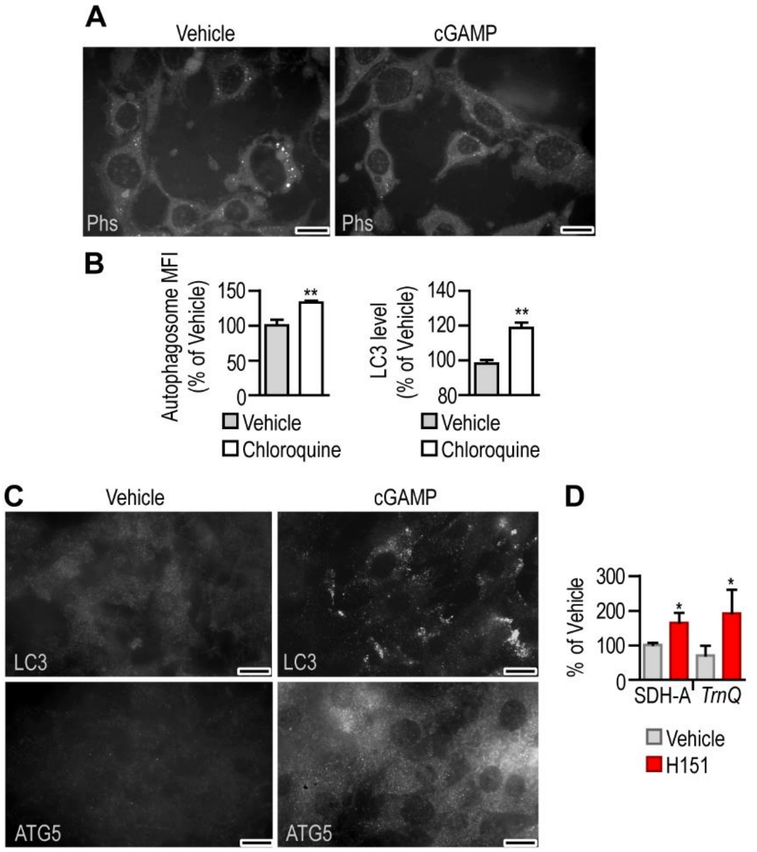

Supplemental Figure 3.

(A) Autophagosomes (Phs) were labeled with a fluorescent probe in BAT-derived mouse adipocytes treated with vehicle or 10 mg/ml cGAMP for 2h. Scale bar 20 µm. (B) *Left:* A fluorescent autophagy assay was used to estimate autophagosome number. Autophagosome mean fluorescence intensity (MFI) in mouse preadipocytes treated with vehicle or 100 µM chloroquine for 4h. *Right:* LC3 level was quantified with an in-cell ELISA in mouse preadipocytes treated with vehicle or 100 µM chloroquine for 4h. (C) LC3 and ATG5 immunostaining of mouse primary preadipocytes treated with vehicle or cGAMP. Corresponding experiment with differentiated adipocytes is shown in Fig. 4D,E. (D) Level of mitochondrial succinate dehydrogenase A protein (SDH-A) and mitochondrially encoded transfer RNA *TrnQ* in mouse preadipocytes treated with vehicle or H151 for 18h. *$p<0.05$, **$p<0.01$, Student's unpaired 2-tailed t-test.
